# Determining adolescent health information needs: A survey in Geneva, 2022

**DOI:** 10.1016/j.pmedr.2024.102682

**Published:** 2024-03-13

**Authors:** Hariel Spinelli Anne-Laure, Morsa Maxime, Baratti-Mayer Denise, Bonvin Nathalie, Soroken Cindy

**Affiliations:** a Geneva University Hospitals (HUG), Geneva, Switzerland; b Psychology Department, Adaptation, Resilience & Changement Unit of Research, University of Liège, Belgium; c Sorbonne North Paris University (LEPS) – Bobigny Campus, Paris, France; d Service de Santé de l'Enfance et de la Jeunesse (SSEJ), Département de Instruction Publique, de la formation et de la jeunesse (DIP), Geneva, Switzerland; e Division of General Pediatrics, Department of Maternity, Adolescent and Child Health, Geneva University Hospitals (HUG), Geneva, Switzerland

**Keywords:** Adolescent, Health information, Health interest, Participatory health research, Need, Public health, Teen

## Abstract

**Purpose:**

When adolescents feel that their opinions are valued and respected, they are more likely to seek and use health services. Our aim was to identify what health information adolescents in Geneva, Switzerland, want and how much they are willing to participate in health promotion, linking it to international recommendations.

**Method:**

Data were collected from 970 adolescents (aged 12–15 years), between September and November 2022, using a classroom-based survey.

**Results:**

Adolescents expressed their preferences: the study showed that the topics of sleep, self-perception, biology, mental health, and physical activity received the highest interest. Girls were more interested than boys in the issues of sleep, mental health, violence, and social life. Adolescents' increasing interest in sexuality issues as they get older is inversely proportional to their interest in other issues such as pandemics, medical research, and the environment, which they are more interested in during early adolescence. Everyone over two felt it was important to promote health in their social environment. And more than a third of teens are open to participating in such activities.

**Discussion:**

Adolescents are open to various health subjects, except for pandemics. Age dependent interest highlights the importance of tailoring intervention programs. Adolescents consider health promotion important and are willing to participate, as recommended by the World Health Organization. By involving them as experts in their adolescence, we have taken an essential step. We strongly recommend continuing involving and trusting adolescents in shaping future interventions.

## 1 Introduction

As chronic diseases and healthcare costs increase, health education, promotion and prevention are major challenges for the future. Adolescents are our future and can be health vectors. The United Nations Secretary-General states that adolescents are “central to everything we want to achieve and to the overall success of the 2030 Agenda”  (World Health Organization, 2017). The World Health Organization (WHO) reminds us that when it comes to adolescent health, the investment is threefold: for their own health in the short term, for their health in adulthood and for the health of future generations  (World Health Organization, 2017).

Public intervention programs might vary in their approach to address the specific interests and concerns of adolescents.

Involving adolescents in decisions that affect their health is not only good practice, but also a right enshrined in the United Nations Convention on the Rights of the Child  (World Health Organization, 2019).

Most reports on health intervention programs rely on epidemiological data and health questionnaires, such as the HBSC questionnaire (Health Behavior in School-aged Children) for young people aged 11 to 15 in 41 countries  (HBSC, 0000, Park et al., 2023). However, to our knowledge, few surveys have directly identified adolescents' interests in health topics. One survey used a text-message method  (Schnall et al., 2013), and two surveys identified these interests directly through a questionnaire, among adolescents aged 14–19 with a focus on gender differences  (Martinović) or students over 18, with an emphasis on health habits, mental health, HIV and sexual transmitted infection prevention  (Vandentorren et al., 2005). However, none of them used a participatory method.

By consulting and observing adolescents, we gain valuable insights into their perspectives, needs and preferences, enabling us to design interventions that are better suited to their specific requirements  (Patton et al., 2016). It was of utmost importance for us to directly consult adolescents because we firmly believe that when their opinions are valued and respected, they are more likely to actively engage with and utilize health services  (World Health Organization, 2017). Our decision to conduct participatory research aligns with the recommendations of WHO, which stress the significance of incorporating adolescents' perspectives and expectations in national health planning processes. This approach ensured that adolescents played an active role in shaping the research and their voices were truly heard.

Involving adolescents in health decision-making can help build trust and a sense of ownership, fostering their empowerment and autonomy. The self-determination theory suggests that an individual's motivation is driven by the satisfaction of psychological needs for autonomy, competence and social interaction  (Deci, 2008). A study examining the motivational factors behind adolescents' propensity to engage in either prosocial or risky behaviors confirmed the importance of autonomous motivation  (Hardy et al., 2015).

Our primary aim was to identify the interests of Geneva adolescents regarding health information to better tailor our intervention programs to their expressed needs. We define interests as a sense of curiosity about what might be in their favor. We will refer to all health prevention, promotion and education programs as intervention programs. Our secondary objective was to assess their willingness to participate as health promoters.

## 2 Methods

We conducted an exploratory quantitative study.

### 2.1 Participatory approach

From the beginning, we included adolescent partners in our working group. Through their active participation, the topics and questions for the survey were identified.

In September 2021, we recruited five adolescents' volunteers, from the local middle school through a teacher we knew, simply by inviting them to help us design the questionnaire. We organized three working sessions from September to November 2021. During these sessions, adolescents identified relevant topics of interest and provided guidance on survey distribution. We then designed and refined the questionnaire with their input.

In April 2022, we tested the questionnaire with a different group of adolescents recruited from both outpatient adolescent units and adolescents hospitalized in pediatrics at that time. These adolescents met the inclusion criteria for the study and where in a health condition that allowed them to comfortably participate in the questionnaire test. This was done to ensure the effectiveness and representativeness of the questionnaire. Following feedback and adjustments, we finalized the questionnaire using Google Forms® software, while also preparing a paper version for participants without access to a computer.

### 2.2 Participants

Considering the organizational structure of the Swiss school system, which brings together all middle school students (the US 6th, 7th and 8th grades) in a single institution, we made a strategic decision to initially focus within this age group of 12 to 15 years old.

We included adolescents from all social backgrounds, regardless of their health status, attending all levels of education in middle schools of Geneva, Switzerland, both girls and boys. Non-French-speaking adolescents were excluded.

### 2.3 Recruitment

Given a total of 14,785 registered middle school students in Geneva (13,795 in public and 990 in private schools), the recommended minimum sample size (using a tool from the Survey Monkey website) was of 577, with a confidence level of 95 % and a margin of error of 4 %.

In collaboration with the Department of Public Instruction of the Canton of Geneva, we sent an information letter at the end of summer 2022 to the deans of the middle schools, inviting them to participate in the survey on a voluntary basis.

We recruited four middle schools (three public and one private).

A document was sent to parents to inform them about the study even though due to the presumed adolescents' capacity for discernment at this age according to the swiss legislation, we did not need to obtain parents’ explicit consent. Adolescents independently decide whether they wanted to participate or not. Additionally, we created a separate document to inform the teachers about the study and provided them with a comprehensive manual containing specific instructions on how to conduct the survey adequately under their guidance.

### 2.4 The procedure

The questionnaire was anonymized using either online Google Forms® with a QR code for anonymous access or an anonymized paper-based format. It was distributed in French from 09/13/2022 to 11/28/2022.

In two public schools and the private school, the survey was distributed to all teachers, and in the third public school, it was distributed to one teacher at each grade. The paper questionnaires were administered by sworn administrators and by the principal investigator.

### 2.5 Study measures

We created a comprehensive 79-item questionnaire covering 15 topics: *Self-perception*, *Mental health*, *Addictions*, *Safety and Risk Taking*, *Sleep*, *Biology*, *Sexuality*, *Physical activity*, *Food*, *Pandemics*, *Violence*, *The Environment*, *Natural Medicine*, *Research* and *Social Aspects* (cf. **Appendix**).

Each topic was accompanied by a short explanation of the field concerned. For each topic, participants were asked to answer the following question: “In the field of […] I would like to be better informed about: […]”. Questions relating to these themes were then asked. Participants could answer using a Likert scale; the answer options were “no”, “rather no”, “rather yes”, “yes”. They were also asked about their three favorite topics, which they were asked to rank in order of preference. They could add topics they had not found in the questionnaire.

Participants rated the importance of health promotion within their social circles on a scale of 1 to 10 (from “1 = not at all important” to “10 = very important”), as well as their readiness to engage in health promotion, using the same scale (from “1 = not at all” to “10 = very ready”).

Then the questioner ended with a few epidemiologic questions (age, gender identity and the number of family members).

### 2.6 Analysis

We considered statistically significant results with a threshold of p < 0.001.

We calculated summary scores for each of these topics based on the mean score of the relevant items and obtained internal consistency coefficients (Cronbach’s alpha) to verify that these scores were reasonable. We then used analysis of variance, equivalent to a T test for 2 groups comparisons, to compare these scores between different subgroups, including girls vs. boys, different age groups (from 12 to 15, by year), and public vs. private school attendees. For age groups, we obtained a P value for the linear trend. Analyses were done using SPSS version 28®.

### 2.7 Ethics

In Switzerland, the law on research with humans is focused on informed consent regarding disease and the normal functioning of the human body. Since this study does not concern disease, it was considered to fall outside the scope of Swiss human research legislation and received an exemption from the Swiss Ethics Committee. Nevertheless, we provided adolescents with a detailed explanation of the study, and they were given the time and freedom to decide whether to participate. They could at any time stop the survey.

## 3 Results

We collected data from 970 respondents on their information interests; of whom seven failed to answer any questions and were excluded from further analysis. Most of the respondents (797; 82.2 %), answered the survey online, while 173 (17.8 %) provided paper-based responses. Due to the design of the survey, we cannot determine how many invited adolescents actually participated.

Most respondents (86.4 %) reported plausible ages between 10 and 16, while 3.3 % reported non-plausible responses such as 1 to 9 or 17 to 99, and 10.3 % did not answer.

Almost all participants answered the questionnaire very seriously with consistent answers, but a small percentage of adolescents responded in a playful way when asked about their age (2.1 %, with 10.3 % not answering) and gender (6.61 %). We decided to exclude them only for the age and gender subcategory analyses.

In addition, due to a low representativeness, the subgroup of “other” gender was not analyzed as such to see if there were differences in their demand for health information.

Self-identification results were as follows: 47.2 % identified as female, 39.2 % as male, 9.4 % as other and 3.9 % chose not to answer. The “other” responses included several that can be consider valid — such as “gender fluid” (N = 5), “questioning” (N = 1), “not sure” (N = 1), “I don’t know” (N = 3), “child” (N = 4), “non-binary” (N = 8), “no sex” (N = 1), “trans” (N = 1) —, some that informed of sexual preference rather than gender — “Gay” (N = 1), “Lesbian” (N = 2) —, but the majority were fanciful (“Airbus A380”, “Ninja turtle”, “robot”, “shopping bag”, “Shrek”, “toaster”, “unicorn”, etc.). The humorous responses for gender were excluded.

The distributions of the answers across the 79 information items ranged across the 4 options (**Appendix**) and mean values on the 1–4 scale were between 1.81 and 2.89. For all 15 information topics, the corresponding items were moderately to strongly correlated, and as a result, the Cronbach’s alpha coefficients were reasonably high (Table 1), making it possible to analyze the responses to the survey primarily based on the 15 topics studied, rather than analyzing the 79 items separately, justifying the computation of domain-specific summary scores. We confirm that the questions asked were not the cause of the results obtained, as the items and preferred topics were consistent with the averages.

**Table 1:** Dimension-specific scales, among 963 Adolescents, in Geneva, 2022.

	**N items**	**Missing, N**	**Cronbach’s alpha**	Mean* (SD)	**Proportion as first priority**	**Proportion as any priority (1**–**2-3)**
Perception of self	5	12	0.91	2.69 (0.97)	12.5 %	20.4 %
Mental health	6	14	0.83	2.60 (0.88)	12.2 %	24.5 %
Sexuality	11	21	0.94	2.31 (1.00)	9.7 %	23.4 %
Sleep	4	19	0.81	2.67 (0.98)	9.4 %	29.0 %
Physical activity	4	23	0.87	2.60 (1.04)	7.6 %	19.7 %
Addictions	4	20	0.81	2.25 (0.99)	7.5 %	18.1 %
Social life	6	33	0.89	2.53 (0.98)	5.8 %	17.1 %
Diet	7	19	0.88	2.50 (0.94)	5.1 %	20.7 %
Violence	8	21	0.93	2.42 (1.03)	4.0 %	17.0 %
Biology	3	29	0.83	2.64 (1.03)	2.0 %	5.7 %
Medical research	4	35	0.85	2.42 (1.00)	1.8 %	6.6 %
Safety and risk-taking	5	19	0.77	2.28 (0.86)	1.6 %	6.6 %
Environment	5	31	0.89	2.49 (1.02)	1.6 %	7.1 %
Alternative medicines	3	37	0.90	2.36 (1.10)	1.2 %	5.6 %
Pandemics	4	28	0.84	2.04 (0.95)	0.6 %	2.4 %

*Scale between No (1) and Yes (4).

The highest average score was for *Self-Perception* (2.69, p < 0.001), and the lowest was for *Pandemics* (2.04, p < 0.001) (Table 1).

Among the 970 respondents, 168 (17.3 %) did not select any priority area and were not included in the following analyses (Table 1).

No dominant theme was identified, but we did observe that *sleep*, *mental health* and *sexuality* were mentioned among the three priorities. *Sleep* scored highest with 29 % of responses, followed by *mental health* and *sexuality* (Table 1). Girls generally expressed slightly higher information needs than boys (Table 2), with the most marked differences concerning *violence* (2.63, p < 0.001), *sleep* (2.81, p < 0.001)*, social life* (2.65, p < 0.001) and *mental health* (2.72, p < 0.001).

**Table 2:** Adolescent health Information Needs: Subgroup Comparisons (Mean Scores) in Geneva, 2022.

		Gender				Age (years)				School system	
	**Girls**	**Boys**	**P**	**12**	**13**	**14**	**15**	P*	**Public**	**Private**	**P**
Perception of self	2.75	2.71	0.15	2.79	2.70	2.59	2.90	0.41	2.76	2.50	<0.001
Mental health	2.72	2.62	<0.001	2.68	2.63	2.60	2.65	0.53	2.62	2.55	0.28
Addictions	2.27	2.24	0.43	2.29	2.23	2.22	2.34	0.88	2.19	2.38	0.009
Safety and risk-taking	2.30	2.29	0.68	2.44	2.28	2.18	2.31	0.014	2.31	2.21	0.10
Sleep	2.81	2.68	<0.001	2.67	2.77	2.63	2.63	0.35	2.67	2.65	0.79
Biology	2.68	2.65	0.32	2.69	2.73	2.55	2.51	0.049	2.66	2.57	0.24
Sexuality/reproduction	2.35	2.28	0.028	2.14	2.24	2.42	2.48	<0.001	2.29	2.34	0.56
Physical activity	2.53	2.61	0.009	2.67	2.64	2.58	2.48	0.10	2.64	2.49	0.042
Diet	2.59	2.51	0.004	2.58	2.54	2.45	2.58	0.32	2.53	2.42	0.13
Pandemics	2.02	2.04	0.45	2.20	2.10	1.93	1.90	<0.001	2.07	1.98	0.24
Violence	2.63	2.43	<0.001	2.44	2.37	2.45	2.54	0.31	2.40	2.46	0.41
Environment	2.57	2.50	0.032	2.69	2.53	2.41	2.24	<0.001	2.51	2.45	0.47
Alternative medicines	2.42	2.36	0.11	2.41	2.47	2.36	2.11	0.07	2.36	2.35	0.86
Medical research	2.45	2.43	0.60	2.58	2.54	2.31	2.21	<0.001	2.43	2.40	0.68
Social life	2.65	2.54	<0.001	2.68	2.59	2.50	2.54	0.08	2.54	2.51	0.66

*P for linear trend.

**Fig. 1:**
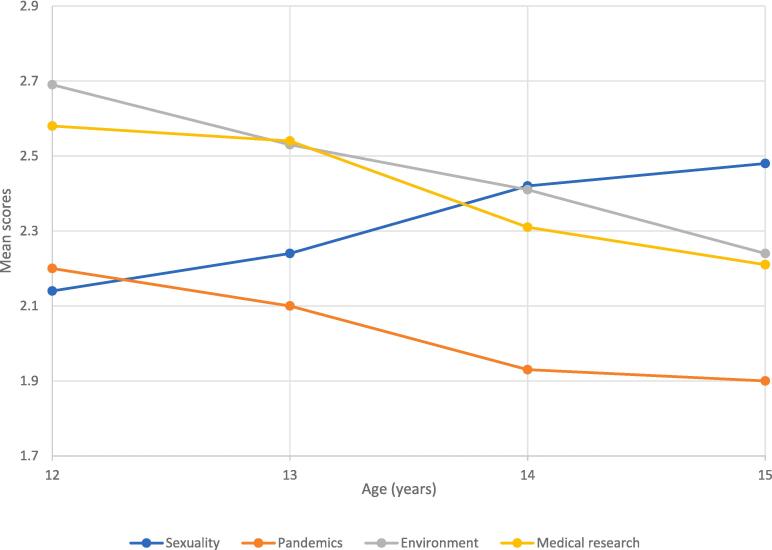
Significant Health Interest Evolution Across Adolescent Age Groups in Geneva, 2022.

Interest in *sexuality* increased with age, while the opposite is observed for *the environment*, *medical research* and *pandemics* (Table 2 and Fig. 1).

Most respondents (71.9 %) attended public school. Public and private school students expressed similar information needs for most topics, except for *Self-perception* where private school students expressed lower needs and higher needs for *Addictions* (Table 2).

**Fig. 2:**
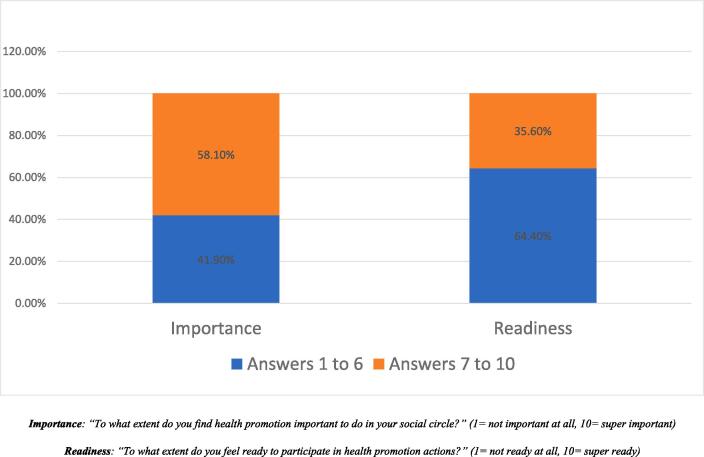
Importance of Health Promotion and Participation Readiness Among Adolescents in Geneva, 2022.

With regard to the question “How important you think it is to promote health in your social circle?”, the average score was 6.7, with a standard deviation of 2.7. In addition, 58.09 % of respondents rated health promotion as important to very important (7–10 on the scale) (Fig. 2).

As for the question: “To what extent do you feel ready to participate in health promotion actions?”, the average score was 5.4, with a standard deviation of 2.7. The proportion of participants scoring between 7 and 10 was 35.58 % (Fig. 2).

## 4 Discussion

The survey achieved a high response rate, with most adolescents actively participating and providing answers to most of the questions. This indicates their interest and willingness to engage in the survey. While no single topic emerged as dominant, the results revealed that adolescents expressed a broad range of interests in various health topics, except for pandemics. The lower interest in this subject could suggest a sense of exasperation or the perception that they already possess enough information on it due to the recent experience with COVID-19.

The study shows that the topics of *mental health*, *self-perception*, *sleep* and *sexuality* generated the highest level of interest and were also identified as priorities by adolescents.

Adolescents prioritize *mental health*, which is not surprising given its significant impact. More than half of mental disorders emerge during adolescence  (García-Carrión et al., 2019), affecting 1 in 7 individuals aged 10–19 globally. Factors like family breakdown, academic and occupational pressures contribute to the rising prevalence of mental health difficulties among young people. The COVID-19 pandemic has worsened these challenges by reducing protective factors such as routines and social connections  (Alamolhoda et al., 2023, Amran, 2022). Also, adolescents show a heightened sensitivity to the suffering of their peers and frequently exhibit a tendency to mimic their emotions and behaviors  (Laursen and Veenstra, 2021). They are profoundly affected by the challenges faced by their friends and may mirror their emotional states and actions. This increased exposure to the struggles of their peers drives them to actively seek information, aiming to gain a better understanding of mental health issues and how to effectively support their friends. Their motivation lies in providing the best possible assistance and support to their peers in need, based on our clinical experience.

In our survey, teenagers exhibited a strong interest in self-perception. Self-esteem assumes a pivotal role during this developmental stage, serving as a robust predictor of peer relationships, psychological well-being, and future success. On average, self-esteem tends to rise with age in adolescence  (Orth, 2017). While the specific factors driving this heightened interest remain unclear to us, we believe adolescents seek resources to effectively navigate challenges in areas such as social relationships, identity, and sexuality. Considering the substantial impact of adolescent self-esteem on adult life, further exploration could facilitate the development of targeted interventions to enhance overall life satisfaction.

Sleep problems among adolescents have become increasingly prevalent worldwide  (Norell-Clarke and Hagquist, 2017, Twenge et al., 2017). Adolescents may experience sleep difficulties due to various reasons, including stress, school pressures, unhealthy sleep habits, existential thoughts and mental or physical suffering  (Jakobsson et al., 2019, Jakobsson et al., 2020). By taking a holistic approach and considering these multifaceted influences, tailored interventions can be developed to promote healthy sleep habits and improve overall well-being in this population.

There are gender, age and school environmental differences. Girls are more interested in *sleep*, *mental health*, *violence* and *social life*. In addition, *sleep* was a priority for 29 % of teens, with girls showing more interest in this topic than boys. Gender-based differences were also documented in another European country (Croatia) by Martinovic et al.  (Martinović et al., 2022). Tailoring health intervention programs with a gender focus and promoting awareness of each other's concerns can be a challenging task for professionals. It can contribute to more equitable and effective healthcare practices.

The success of prevention and intervention programs largely depends on implementing them at the right time and tailoring their content to specific needs  (Who, 2018). For instance, studies have shown that addressing sexuality issues before adolescence can have a more significant impact  (Breuner, et al., 2016, Standards for Sexuality Education in Europe, 2011). Our participants showed an increasing interest in issues related to sexuality as they get older. During adolescence, there is a natural progression with significant increase in sexual curiosity and interest (including sexual desire, sexual arousal, sexual behaviors and sexual function)  (Breuner, et al., 2016). Adolescents may start exploring their sexual identities, developing romantic and intimate relationships, and engaging in more complex sexual behaviors. By recognizing the continuum of sexuality development, intervention programs can be tailored to specific age groups, ensuring that the content is relevant and meaningful to adolescents at different stages of their development. This approach acknowledges the unique needs and concerns of adolescents, promoting informed decision-making, healthy relationships, safe sexual practices and maintaining their overall well-being. We suggest revising intervention programs to specific age groups and reinforcing messages about sexuality as adolescents mature.

Additionally, it may be beneficial to engage adolescents around the age of 12 in discussions about topics such as the *environment* and *medical research*, as interest tends to be high at this age. Just as with older adolescents, who often exhibit declining interest in these subjects, there is a challenge in exploring effective strategies to maintain their interest. Our study suggests the advantages of introducing these topics early, which can foster a deeper understanding and promote responsible decision-making. This approach ensures ongoing relevance and engagement with these important issues.

Students in the public and private school systems expressed similar information needs, except for private school attendees who expressed lower needs for *Self-Perception* but higher needs for *Addictions*. While individual factors contribute to substance abuse, the school culture and environment can play a role in shaping adolescent behaviors. In Geneva, Switzerland, private schools, frequently attended by affluent students, might cultivate an environment where substance use is prevalent. Affluence could facilitate easier access to substances, while social dynamics may normalize their usage. This heightened interest among private school students underscores the importance of additional research to grasp the issue comprehensively and implement effective interventions, aligning with our clinical insights.

This study is the first to find out directly from adolescents in Geneva what health information topics they are interested in, especially using a participatory approach. First, because we did not want to miss any important topic at their eyes. Second, with this research, we wanted to concretely promote adolescents' participation in health actions in a pragmatic way: producing knowledge for concrete action.

Although adolescents are known to emulate their peers and are prone to engaging in risky behaviors, they also emulate prosocial behaviors, which may encourage their participation in public health initiatives  (Andrews et al., 2020). Therefore, peer participation in public health campaigns is an important motivator for adolescents, more so than for adults  (Foulkes et al., 2018). It is worth noting that although adolescents' imitation of peers is most pronounced in their presence, it also continues, albeit to a lesser extent, in their absence  (Choukas-Bradley et al., 2015). Furthermore, interventions tailored to adolescents' respect, autonomy, and values tend to be more effective than those solely designed and delivered by adults  (Yeager et al., 2018).

Our study found that just over one third of participants were receptive to participating in health education initiatives. This finding is significant according to self-determination theory  (Deci, 2008). Overall, more than one over two adolescents found it important to very important to promote health in their family, friends and community. This finding highlights the tremendous potential to improve the health and well-being of the community.

As healthcare providers, it is essential that we acknowledge and embrace the engagement of our adolescents. We must be proactive in responding to and directing this positive energy.

### 4.1 Limitations and bias of the study

Our presence during the working sessions may have influenced the adolescents' level of spontaneity, which may have affected their responses.

Additionally, our adolescent partners and participating schools were volunteers, which could introduce selection bias and limit the generalizability of our findings to the wider adolescent population.

Another limitation is the lack of information on participants' sources of information, such as family, parents, school, friends or social networks, which could have provided insights into their level of interest. The restrictive nature of our questions prevented us from analyzing these aspects in depth.

To minimize bias, multiple evaluators assessed the data, which helped mitigate potential halo effects.

### 4.2 Perspectives

Adolescence, encompassing ages 10–24  (Sawyer et al., 2018), involves significant brain development and behavioral, emotional, and cognitive changes. Effective interventions should align with developmental stages, addressing for example early adolescence's risk behaviors and later adolescence's mental health challenges. Adapting health education to this evolving understanding is crucial. We plan to repeat the survey with adolescents aged 16–19 years to see similarities or differences and to better target intervention programs.

## 5 Conclusion

According to current international recommendations, we directly consulted adolescents aged 12 to 15 in Geneva to understand their health information needs. Interestingly, no dominant choice emerged. Our survey revealed that adolescents are open to a wide range of health topics, except for the subject of pandemics.

However, we did observe a growing interest in sexuality as they progress through puberty. Additionally, younger adolescents showed a heightened sensitivity to information related to environmental issues and medical research. Therefore, it is crucial to consider the timing and relevance of intervention programs, considering the different stages of development, including the exploration of sexuality and the importance of environmental awareness.

Lastly, our survey revealed that one third of the participants expressed a strong desire to actively participate in health-related initiatives because they perceive it as significant. This emphasizes the importance of trusting and involving adolescents in shaping future interventions. By engaging them as partners, we can harness their perspectives and empower them to contribute to decision-making processes, ensuring that our health initiatives are relevant and meaningful to their needs.

## 6 Implications and contribution

- Involving adolescents in decisions that affect their health is not only good practice, but also their right.
- By consulting adolescents, we can better understand their needs and preferences and tailor our interventions to meet their specific needs. Just ask them!

## CRediT authorship contribution statement

**Hariel Spinelli Anne-Laure:** Conceptualization, Methodology, Formal analysis, Investigation, Resources, Writing – original draft, Writing – review & editing, Validation, Supervision, Project administration. **Morsa Maxime:** Conceptualization, Methodology, Writing – review & editing, Formal analysis, Validation, Supervision. **Baratti-Mayer Denise:** Conceptualization, Writing – review & editing, Formal analysis. **Bonvin Nathalie:** Conceptualization, Writing – review & editing, Formal analysis. **Soroken Cindy:** Conceptualization, Methodology, Formal analysis, Investigation, Resources, Writing – original draft, Writing – review & editing, Validation, Supervision, Project administration.

## Funding

There is no funding declared.

## Declaration of competing interest

The authors declare that they have no known competing financial interests or personal relationships that could have appeared to influence the work reported in this paper.

## Data Availability

Data will be made available on request.
