# Supplementary material for: Determining adolescent health information needs: A survey in Geneva, 2022
Source: Prev Med Rep. 2024 Mar 13;41:102682. doi: 10.1016/j.pmedr.2024.102682 (PMC10959694; doi:10.1016/j.pmedr.2024.102682)
Supplement: Supplementary data 1 [file mmc1.docx]

# Appendix: ADOLESCENTS’ QUESTIONNAIRE, GENEVA, 2022 – WHAT HEALTH INFOrmation interest you?

Good morning,

With this questionnaire, we aim to understand your information needs related to various health topics.

**How much information would you like to receive about the following health topics?**

WARNING: If you are answering this questionnaire on your phone, please click on each arrow next to each theme (on the right side of your screen) to display the descriptions and the available answer choices.

## 1: My perception of myself

Self-esteem is a person's perception of their own worth and their ability to value their qualities and personal growth, according to the World Health Organization (WHO). A good self-esteem helps develop a sense of security and promotes a positive self-image.

Concerning the perception I have of myself, I would like to be more informed about:

|  | **No** | **Rather no** | **Rather yes** | **Yes** |
| --- | --- | --- | --- | --- |
| Self-confidence |  |  |  |  |
| Self-affirmation |  |  |  |  |
| Self-acceptance (of one's differences, disability, etc.) |  |  |  |  |
| Body image |  |  |  |  |
| Self-esteem |  |  |  |  |

## 2: Mental health

Mental health is a state of well-being in which a person manages to overcome life's problems, be productive and relate well to others.

In the field of mental health, I would like to be more informed about:

|  | **No** | **Rather no** | **Rather yes** | **Yes** |
| --- | --- | --- | --- | --- |
| The importance of having social or friendly relationships and their benefits on our health |  |  |  |  |
| Stress and pressure management |  |  |  |  |
| The normal mood fluctuation and when to worry |  |  |  |  |
| Mental illnesses (depression, post-traumatic stress, bipolarity, schizophrenia, etc.) |  |  |  |  |
| Treatment of mental illnesses |  |  |  |  |
| The prevention of mental illnesses (detecting the warning signs, knowing the available resources, etc.) |  |  |  |  |

## 3: Addictions

I love you a little, a lot, passionately... madly? According to the WHO, addiction is a state of short- or long-term dependence on substances (alcohol, tobacco, etc.) or behaviors (games, screens, etc.).

In the field of addictions, I would like to be more informed about:

|  | **No** | **Rather no** | **Rather yes** | **Yes** |
| --- | --- | --- | --- | --- |
| The risks associated with excessive alcohol consumption |  |  |  |  |
| The risks associated with excessive tobacco consumption (cigarettes, electronic cigarettes) |  |  |  |  |
| The risks associated with the excessive consumption of other substances (cannabis, cocaine, LSD, etc.) |  |  |  |  |
| The risks associated with excessive use of screens (phones, game consoles, etc.) |  |  |  |  |

## 4: Safety and risk taking

This theme concerns our behavior when we are perpetrators, witnesses or victims of dangerous situations, both for ourselves and for others. This theme does not address the risks related to addictions and sexuality which are covered in other sections.

In the field of safety and risk-taking, I would like to be more informed about:

|  | **No** | **Rather no** | **Rather yes** | **Yes** |
| --- | --- | --- | --- | --- |
| Capable or not capable challenges |  |  |  |  |
| What to do and who to contact in an accident situation (first aid training) |  |  |  |  |
| The risks of dangerous driving (non-compliance with the highway code: speed, seat belt, etc.) |  |  |  |  |
| The risks of doing acrobatics with or without a vehicle: chases, street performances, .etc. |  |  |  |  |

## 5: The sleep

This theme concerns sleep in general (dreams, snoring...), its disorders (insomnia...) and its disturbing factors (noise, light, temperature,..).

In the field of sleep, I would like to be more informed about:

|  | **No** | **Rather no** | **Rather yes** | **Yes** |
| --- | --- | --- | --- | --- |
| The stages of sleep (light, deep and paradoxical) |  |  |  |  |
| Recommendations for sleeping well |  |  |  |  |
| The consequences of a lack of sleep |  |  |  |  |
| Sleep disorders (difficulty falling asleep, nocturnal awakenings, nightmares, sleepwalking, etc.) |  |  |  |  |

## 6: Biology

This theme concerns the functioning and properties of the organs and systems of the human body when it is healthy or ill.

In the field of biology, I would like to be more informed about:

|  | **No** | **Rather no** | **Rather yes** | **Yes** |
| --- | --- | --- | --- | --- |
| What happens when the human body is healthy (breathing, digestion, blood vessels, …) |  |  |  |  |
| What happens when the human body is unhealthy / sick (problem with breathing, digestion, blood vessels, …) |  |  |  |  |
| the signals to spot when the body has an activity that is not normal |  |  |  |  |

## 7: Sexuality

According to the WHO, sexuality is defined as a central aspect of human life which includes in particular intimacy, pleasure, desire, sexual orientation, ... Sexuality can be influenced by different things: biology, psychology, culture, religion, relationships, etc.

In the field of sexuality, I would like to be more informed about:

|  | **No** | **Rather no** | **Rather yes** | **Yes** |
| --- | --- | --- | --- | --- |
| Puberty (change in genitals, erection, masturbation, vaginal discharge, change in voice, appearance of body hair, menstruation, etc.) |  |  |  |  |
| Romantic and/or sexual attraction |  |  |  |  |
| Gender identity (identifying as male, female, neither, both at the same time, etc.) |  |  |  |  |
| Sexual relations (age of sexual majority, pleasure, consent, respect, sexual violence, etc.) |  |  |  |  |
| Contraception (condom, pill, intrauterine device, etc.) |  |  |  |  |
| Emergency contraception (pill to be taken after unprotected sex with risk of pregnancy) |  |  |  |  |
| Pregnancy (how to get pregnant, symptoms, hormones, effects on the body...) |  |  |  |  |
| Abortion or termination of pregnancy |  |  |  |  |
| Sexually transmitted infections (gonococcus, chlamydia, AIDS, papillomavirus, etc.) |  |  |  |  |
| Pornography |  |  |  |  |
| What does the law say about pregnancy (right to have children, age of sexual majority, etc.) |  |  |  |  |

## 8: Physical activity

This theme concerns the health effects of all movements that are carried out in the context of leisure, at school or to move from one place to another.

In the field of physical activity, I would like to be more informed about:

|  | **No** | **Rather no** | **Rather yes** | **Yes** |
| --- | --- | --- | --- | --- |
| The harms of lack of physical activity (sedentary lifestyle) |  |  |  |  |
| The health benefits of moving/having physical activity |  |  |  |  |
| Daily physical activity recommendations |  |  |  |  |
| The harms of excessive sports activity |  |  |  |  |

## 9: Food

This theme includes everything related to food and diets: the composition of meals, recommendations, intolerances, bad eating habits, etc.

In the field of food, I would like to be more informed about:

|  | **No** | **Rather no** | **Rather yes** | **Yes** |
| --- | --- | --- | --- | --- |
| How to have a healthy and balanced diet: carbohydrates (sugars), lipids (fats), proteins |  |  |  |  |
| The misdeeds of junk-food: fast-food, industrial products and additives (biscuits, frozen pizzas, ready meals, hidden sugars, etc.) |  |  |  |  |
| the signs to spot when you are hungry and when you have eaten enough |  |  |  |  |
| Diets (vegetarian, vegan, lactose-free, gluten-free, etc.) |  |  |  |  |
| Food intolerances and allergies |  |  |  |  |
| Difficulties around how to eat |  |  |  |  |
| Personal healthy body weight |  |  |  |  |

## 10: Pandemics

According to the WHO, a pandemic is the global spread of a new disease. This theme includes everything related to pandemics (COVID-19 and others), research, viruses, vaccines, etc.

In the area of pandemics, I would like to be more informed about:

|  | **No** | **Rather no** | **Rather yes** | **Yes** |
| --- | --- | --- | --- | --- |
| The various past pandemics (COVID-19, AIDS, flu, plague, cholera): figures, symptoms, duration, sequelae, ... |  |  |  |  |
| The health pass |  |  |  |  |
| Sanitary measures (lockdown, mask-wearing, social distancing, etc.) |  |  |  |  |
| Vaccines (types, effectiveness, etc.) |  |  |  |  |

## 11: Violence

This theme refers to all types and all forms of violence regardless of the environment (school, family, social networks, community, etc.) or age and whether one is the perpetrator, witness or victim.

In the field of violence, I would like to be more informed about:

|  | **No** | **Rather no** | **Rather yes** | **Yes** |
| --- | --- | --- | --- | --- |
| Physical or verbal abuse |  |  |  |  |
| Sexual violence (sexual assault, rape, etc.) |  |  |  |  |
| Incest (sexual activity between members of the same family) |  |  |  |  |
| Harassment (school, family, ...) |  |  |  |  |
| Cyberbullying (social networks, sexting, etc.) |  |  |  |  |
| The short and long-term consequences on the victims, witnesses and perpetrators of violence (vision of oneself, self-esteem, fear, shame, regrets, etc.) |  |  |  |  |
| How to react when you are a victim, witness or perpetrator of violence |  |  |  |  |
| Violence against oneself (self-harm) |  |  |  |  |

## 12: The environment

This theme concerns the impact of our external environment on human health: pollution, pesticides, climate change, hormonal disruptors, etc.

In the field of the environment, I would like to be more informed about:

|  | **No** | **Rather no** | **Rather yes** | **Yes** |
| --- | --- | --- | --- | --- |
| The health problems that our environment can cause (pollution, pesticides, hormone disruptors, etc.) |  |  |  |  |
| Global warming and its consequences on health |  |  |  |  |
| The significant anxiety and the feeling of helplessness felt in the face of climate change |  |  |  |  |
| How to avoid environmental degradation to protect our health |  |  |  |  |
| What will our food look like in the future? |  |  |  |  |

## 13: Natural medicine

This theme brings together all the therapeutic approaches that are different from the medicine that we are used to knowing. It deals in particular with natural, unconventional and alternative medicine.

In the field of natural medicine, I would like to be more informed about:

|  | **No** | **Rather no** | **Rather yes** | **Yes** |
| --- | --- | --- | --- | --- |
| The other types of medicine that exist (homeopathy, plants, acupuncture, hypnosis, etc.) |  |  |  |  |
| The proven effectiveness of these other types of medicine |  |  |  |  |
| The complementarity of conventional medicine and other medicines |  |  |  |  |

## 14: Medical research

This theme covers all activities aimed at acquiring knowledge about a health problem or finding a possible solution to it. Research helps scientists formulate prevention strategies, new treatments, or new health care practices.

In the field of research, I would like to be more informed about:

|  | **No** | **Rather no** | **Rather yes** | **Yes** |
| --- | --- | --- | --- | --- |
| How to conduct medical research |  |  |  |  |
| The concrete achievements of medical research |  |  |  |  |
| How to recognize reliable information and false information |  |  |  |  |
| Medical research on animals |  |  |  |  |

## 15: The social

This theme relates to the influence of social (relationships with others, culture, disability, etc.) and economic (work, income, unemployment, etc.) factors on health.

In the social field, I would like to be more informed about:

|  | **No** | **Rather no** | **Rather yes** | **Yes** |
| --- | --- | --- | --- | --- |
| How social and economic factors can influence family functioning |  |  |  |  |
| How the lack of money can impact health (problems with food, housing, ...) |  |  |  |  |
| How being from a foreign culture can impact health |  |  |  |  |
| How having a sick or disabled loved one can impact health |  |  |  |  |
| How to behave with people with disabilities |  |  |  |  |
| How to have good relations with those around me |  |  |  |  |

## 16. What information media(s) do you use?

(Several answers possible) Multiple choice.

- TikTok
- Instagram
- Facebook
- Podcasts/Youtubers
- Websites
- Magazines
- I don't use it
- Other : …

## 17. On which media(s) would you like to receive health information?

(Several answers possible) Multiple choice.

- TikTok
- Instagram
- Facebook
- Podcasts/Youtubers
- Websites
- Magazines
- Other : …

## 18. To what extent do you feel ready to participate in health promotion actions?

(1 = Not at all ready, 10 = Very ready!)

- 1
- 2
- 3
- 4
- 5
- 6
- 7
- 8
- 9
- 10

## 19. To what extent do you find it important to promote health around you (family, friends, entourage)?

(1 = Not at all important, 10 = Super important)

- 1
- 2
- 3
- 4
- 5
- 6
- 7
- 8
- 9
- 10

## 20. Among the themes presented, what are your 3 favorites?

(Write them in order of preference starting with your favorite)

1 - My perception of myself

2 - Mental health

3 - Addictions

4 - Safety and risk taking

5 - Sleep

6 - Biology

7 - Sexuality

8 - Physical activity

9 - Food

10 - Pandemics

11 - Violence

12 - The environment

13 - Natural medicine

14 - Medical research

15 - The social

Enter your answer: …

## 21. On what other topic(s) would you like to receive health information?

Enter your answer: …

**To CONCLUDE...**

We are going to end this questionnaire by asking some information about you, which will of course remain anonymous:

## 22. How old are you?

Enter your answer: …

## 23. What is your gender identity?

(Gender felt, with which you identify) Single choice.

- A woman
- A man
- Non-binary
- Gender-fluid
- Other : …

## 24. How many people live with you at home?

(Not counting you) Single choice.

- One
- Two
- Three
- Four and more

**Thanks! Your answers will be taken into account!**
